# The Institutional Worker

**Published:** 1913-01-25

**Authors:** 


THE
### INSTITUTIONAL WORKER
Being a Special Supplement to "The Hospital"

### BUREAU OF INFORMATION.

### Rules for Correspondents.

1. Every letter, on whatever subject, must be accompanied by the coupon to be cut from the back cover (inside page) of The Hospital, current issue, and must contain the name and address of the correspondent with pseudonym for publication if desired.

2. Replies by post cannot be given, save under exceptional circumstances at the Editor's discretion.

3. Letters from Approved Homes in reply to special needs published in the Bureau should state terms and full particulars, and be sent prepaid under cover to the Editor of the Bureau with name written across coupon for identification.

4. Proprietors of Homes which have not yet been entered on the List of Approved Homes, but have spare accommodation likely to suit special needs, are invited to write for an application form for registration. The fee for registration, which includes two announcements of the Home in the Bureau, is 5s.

5. All communications to be addressed to the Editor of The Hospital, 28 Southampton Street, Strand, London, W.C., and marked "Bureau of Information."

INVITATION TO READERS.—The Editor is prepared to forward to applicants, without fee on either side, offers of accommodation from Homes on the Approved List suitable for medical and surgical cases, for the aged, for chronic or mental patients, for electrical treatment and massage, for convalescents and for children, at varying terms, in any required locality. Reduced terms can often be arranged. The special needs of any who may be sick or in difficulties are also made known in these columns free of charge.

### Approved Homes Recently Added to List.

Note.—*No Home is added to the List without medical and other testimony to its good management.*

**London, S.E.**—49 Catford Hill. Principal: Mrs. Ambler. Maternity, chronic, and rest-cure cases. Massage if required. Detached house, large and comfortable. Good garden. Open situation.

### Nursing Homes Wanted.

**For Aged Lady.**—The patient is an old lady (not very well off) who requires constant attendance night and day owing to her extreme weakness, though she has no actual disease. She lives entirely on liquid food, and can seldom get up. She is now living alone in rooms with a nurse, but requires more attention than can be given under these circumstances. Locality preferred is South Devon, and, if possible, Dawlish. Great kindness is needed as well as good nursing.—M. C. R. (144a).

**For Elderly Patient, Slightly Mental.**—The patient is an old lady whose mind is slightly affected. A kind, permanent Home is required for her at moderate terms.—J. R. M. (145a).

### Miscellaneous.

**Keeley Institute.**—The Secretary of the Keeley Institute for the Treatment of Inebriety is Mr. Milo H. Aspinwall, and the address is 9 West Bolton Gardens, Earl's Court, London, S.W.—M.

### Needs and Helpers.

**Mission Priest in Transvaal.**—We are sending you this mail full particulars and rules of the Clergy Nursing Home, the Hostel of St. Luke. It is probable that in your circumstances no charge would be made, but you must apply direct to the Secretary, Lieutenant-Colonel C. A. Cooper, 14 Fitzroy Square, London, W. The *x*-ray treatment would, we are informed, have to be given at the specialist's house and at the patient's expense. No doubt the private circumstances would be considered. Will you let us know if we can be of any service on your arrival in recommending rooms or in any other direction?—Rev. F. M. L.

**Motherless Baby.**—Our sympathies have been enlisted on behalf of a baby of seven months whose mother died of eclampsia. The father earns just over £1 a week, and has another child to support, while his wife left him heavily in debt, which he is now paying off. Case [E. London] well recommended by district midwife, to whom letters will be forwarded from any readers who may have it in their power to help.—Nurse Y. Z. (146x).

**Home for Advanced Consumptive.**—We are sorry to hear your sister is not likely to improve. Write to the Sister-in-Charge, St. Michael's Home, Cheddar, and state your circumstances fully, as you have done to us. It is improbable that she has an immediate vacancy, but if the case is thought suitable you could ask to have the name entered, and meantime you cannot do better than keep your sister where she is. We fear admission to the Poor-Law infirmary is the only other alternative.—Night Sister S. W.

### Training and Employment.

**Male Nursing.**—There is very little prospect of getting trained in a civil hospital. But the Army Medical Corps offers great inducements to suitable candidates and an admirable three-years' course of training. As you are the right age and height, with a good education, you would find no difficulty in enlisting for this branch of the Service. Your previous experience and certificate in ambulance work will serve you in good stead.—Favoured.

**Short Training for County Service.**—Yes. There are not a few hospitals, below the first rank, which are willing to receive educated women and give them a year's training in nursing for public purposes. They do not like to make this fact public, as it might prejudice the supply of probationers for their training schools. We are pleased to bring before our readers your desire to obtain one year's training, without paying a fee, for a person anxious to enter county service under the Insurance Act. You will, of course, understand that if your applicant wants to become an Insurance *nurse* three years' training is the least she should aim at. Replies to the Secretary, Hospital Almoners' Council, Denison House, 296 Vauxhall Bridge Road, London, S.W.

### Nursing in the Empire and Abroad.

**Arrival in Rhodesia.**—We advise you to go to the Ladies' Presidential Hostel, Sacs House, Salisbury, on your arrival. You can remain there at moderate charges until you have learned something of the conditions prevailing in the Colony. Have you consulted the Secretary of the Rhodesian Committee, South African Colonisation Society? We strongly recommend you to do so, as this Society can obtain a reduction on the ocean fare and have you met by a representative either at Beira or Cape Town. The address is 23 Army and Navy Mansions, 115 Victoria Street, Westminster.—J. K. K.

**Asylum Posts in the United States.**—We fear you will have considerable difficulty in obtaining the post you require as an asylum matron in the United States. These posts are usually filled by persons who have held office in similar institutions in the country. The only thing you can do is to study the advertisements in the New York papers devoted to asylum interests. Perhaps you would do best to consult Mr. R. D. Roberts, United States Agent, Holyhead, North Wales, on this point. We must warn you that Americans have a preference for candidates trained under their own system, and it may be necessary to hold a subordinate post first out there.—GWALIA.

### Letter-Box.

**Communications have been received and attended to from:**— Nurse M. H., Greasby; Miss O. P., Ealing; Miss E. A. T., Kensington.

**The Editor begs to acknowledge the receipt of communications, with enclosures, from:** — Miss E. Bartholomew, South Kensington; Mrs. Stirling, Musselburgh.

**The Editor is much indebted for testimonials received in response to inquiries from:** — Miss, Scott-Hibbs, Dr. Moon, and Miss Pemberton.

**Reports received from:**—Miss Charlotte Sharman The Orphans' Home, Austral Street, S.E.; The Sister-in-Charge, St. Mary's Home for Children, Buxted, Sussex.

By all means postpone your announcement until it is most likely to be of use to you. January is, as you say, a little early for your locality. Let us know when you wish it to appear.—MISS E. M. W.

We hope you may succeed, and shall be very glad to further your aims as far as possible.—CHRONOMETER.

The Editor is much obliged for the information supplied, which has been forwarded to the right quarter.—SECRETARY, Hostel of St. Luke.

Many thanks for all the fresh circulars forwarded.—EMIGRANTS' INFORMATION OFFICE.

You may rely on finding kindness and comfort in any of the Homes which have offered to receive you at the terms you name, and it appears to be only a question of making a choice.—O. T.

Impossible to forward letter, as rules were not complied with.—MISS HEMSLEY.

Received too late for replies in this issue:—Wanderer; Mrs. Padfield; Sumatra.

### Housekeeping Queries.

**Dirty-looking Table Linen.**—We can sympathise with your disgust at the generally smudgy appearance of the tablecloths when they come from the laundress. It may not be altogether her fault. Linen is apt to lose its good colour after much washing, even under good treatment. We cannot recommend your trying any chemicals to blanch it, as none of these can be used without risk of injuring the fabric. If you can command, however, a moderate-sized grass plat you can bleach the linen in the sun without trouble or danger. Wait till the sun gains its power in the spring and expose your articles for two or three days at a time with the corners weighted down. To produce the best effect the linen should be moistened from time to time from a watering can with water in which borax has been dissolved. This is a good plan also for muddy-looking sheets, and indeed most articles constantly washed in a town laundry and dried in hot-air cupboards are the better for this treatment from time to time.—TIDY ONE.

**Little Fruit Tarts.**—Make some tartlets of short crust. When baked fill with cherries, which can be bought at any good grocer's in tins, stoned, in juice. The juice should be thickened into a syrup and a little added to each tartlet after the fruit has been put in. Bottled gooseberries, skinned grapes, apricots—in fact, any kind of fruit—will look pretty and taste delicious used in this way. This is a supper dish which gives little trouble.—HOME SISTER.

**Snowdon Pudding.**—¼ lb. raisins, ¼ lb. breadcrumbs, 3 oz. suet, 1 oz. ground rice, 3 oz. marmalade, grated rind of one lemon, 2 eggs, ½ gill of milk, 3 oz. castor sugar, pinch of salt. Ornament a greased mould with the raisins, stoned and halved, but not divided; put the crumbs, suet, rice, sugar, and lemon rind into a basin, add the salt, and mix it all well together; then beat in the marmalade, eggs, and milk; put the mixture into the prepared mould, and steam for one and a half hour.—SWEET TOOTH.

**Damp Cupboards.**—You will find it a great help to line your shelves and also the back and the sides of the cupboard with thick brown paper, which may be pasted on. Place occasionally a saucer of lime on each shelf to absorb the damp and leave the doors open for an hour or two when it is warm to admit fresh air. Often the receptacle gets the blame which should be laid on the damp articles placed in it. One or two wet sheets will make everything else damp right through.—SALLY.

**To Clean Holland Blinds.**—As your blinds are only slightly soiled, it is not a difficult matter to improve them, and you could easily make them look quite fresh. Lift them down, lay them and the rollers on a table. Gradually unroll them, dusting them as you do so with a clean soft duster. Each side must be so treated. Then rub them over again in the same way, but using in place of the duster stale crumbs of bread or a stiff dough of flour and water or unbaked bread dough. Rub evenly and lightly, letting each stroke overlap the last one. Then dust with a clean duster and re-hang.—M. W.

### Institution Facts and Figures.

QUESTIONS FOR JANUARY.

1. State the method in use in your institution for making either beef-tea or broth, or both. How much does the liquor work out at per pint respectively? State the price paid for the meat, and what saving, if any, is effected by the use of a stock-pot.

2. What difference could you make in the bill of fare for the staff, irrespective of their numbers, if you were allowed an extra shilling per head per week for their board? The original allowance may be estimated at six, seven, or eight shillings per head. It is desired to demonstrate how much could be obtained in luxuries for the extra shilling.

Either or both of the questions may be answered by each contributor. The best answers will be published, and for each contribution considered by the Editor to be of sufficient interest for publication payment of FIVE SHILLINGS will be made.

The following rules must be observed by contributors to the FACTS AND FIGURES Column:—

1. Answers must be written on one side of the paper. They must bear the name and address of the sender and be accompanied by coupon to be cut from the back cover (inside page) of the current issue of THE HOSPITAL. A pseudonym must be chosen if the name is not to be published.

2. Answers must be addressed to the Editor of THE HOSPITAL, 28-29 Southampton Street, Strand, London, W.C.; they must be marked in the left-hand corner "Facts and Figures," and must be received before the end of the month.

### Editor's Notices.

**Contributions:**

Contributions should be written, or preferably typed, on one side of the paper only, and all articles sent in are accepted upon the distinct understanding that they are forwarded to The Hospital only.

The Editor cannot undertake to return MSS. not used, but when a stamped directed envelope is enclosed the MSS. may be returned if a special request is made.

Accepted articles and paragraphs of news will be paid for after publication at the scale rate.

**Address:**

To prevent delay all contributions and letters on editorial business must be addressed exclusively to the Editor, "The Hospital," 29 Southampton Street, Strand, London, W.C. It is important that this regulation shall be strictly observed.

**Correspondence:**

Correspondence on all subjects is invited. The name and address of correspondents must be given as a guarantee of good faith, but not necessarily for publication.

**Special Articles:**

Special articles are invited, and questions, inquiries, and paragraphs upon all matters relating to the work, administration, and management of general, special, mental, fever, cottage and convalescent hospitals, sanatoria, homes, institutions, societies and organisations for the treatment or care of the sick, injured and dependants of all classes. Every matter affecting the interests and welfare of the staff of all grades working in these institutions will receive special consideration.

**Administrative Medicine, Research and Items of News:**

Special terms will be paid for approved articles on subjects in this wide field by experts who have made some section of it their special study and interest. We wish to give prominence to every movement tending to advance accuracy of diagnosis, efficiency in treatment, and the development of modern methods to eradicate disease and promote the welfare of the suffering.

**A Bureau of Information:**

We invite inquiries and applications for information and help in providing for that numerous class of sufferers who require special care or house-room which they cannot obtain in their own homes or secure for themselves. **We cannot, however, prescribe, or recommend practitioners.**

**Books for Review:**

Publishers are particularly requested to send advance proofs of any new books of importance whenever possible, as the Editor has made arrangements to publish immediate reviews on a new plan.

**Photographs, Plans, Blocks, and Illustrations:**

It is requested that wherever possible MSS. may be accompanied by illustrations in any of the above forms. The name of the sender and of the article to which it belongs should be written on the back of each photograph, drawing, or block for purposes of identification.

**Local Papers:**

Newspapers containing reports or news paragraphs should be marked and addressed to the Sub-Editor.

**The Coupon System:**

A coupon will be found at the bottom of the third inside page of the cover each week. This coupon must be attached to every question or inquiry to which an answer is desired in The Hospital.

### To Secure Appointments.

An increasing number of important and lucrative appointments in all departments of Institutional Life are announced every week in this section of The Hospital. As its name implies, this Supplement is devoted to the interests of the Institutional Worker, consequently every effort is made to secure information for incorporation in this section of Vacancies occurring in the Institutional, Poor-Law, and Municipal Services.

If you cannot find among the announcements a post suited to your experience and capabilities, there is the Appointments Wanted Column, which will enable you to bring your requirements directly to the notice of the Governing Bodies and Controlling Officials of Institutions of every description throughout the country for the small fee of 1s. for 21 words.

This form may be used for small prepaid advertisements, and when filled up should be posted to

*The Manager*, The Hospital,
28 and 29 Southampton Street,
Strand, W.C.

| | | |
|---|---|---|
| | | |
| | | |
| | | |
| | | |
| | | |
| | | |
| | | |

**Subscriptions** may begin at any time, and are payable in advance. Cheques and Post-Office Orders should be crossed London County and Westminster Bank, Covent Garden Branch, and made payable to the Manager of The Hospital.

Rates of Subscription (including Postage).

| *United Kingdom.* | | *Foreign and Colonial.* | |
|---|---|---|---|
| Three Months ... | ... 2s. 0d. | Three Months ... | ... 2s. 6d. |
| Six Months ... | ... 4s. 0d. | Six Months ... | ... 5s. 0d. |
| Twelve Months ... | ... 6s. 6d. | Twelve Months ... | ... 8s. 8d. |

*SUBSCRIPTION FORM.*

*Please send me* The Hospital *for..............months, commencing with your issue of............................, for which please find enclosed.........................*

*Name*......................................................

*Address* ......................................................

......................................................

*Date*....................................

*To* .................................... *Newsagent.*

### Manager's Notices.

**Letters relating to the publication, sale, and advertising departments of "The Hospital" must be addressed to The Manager, "The Hospital," The Hospital Building, 28 and 29 Southampton Street, London, W.C.**

**Advertisements:**

To ensure insertion, all advertisements for The Hospital must reach the Manager not later than Wednesday morning in each week. For scale of charges see pages iii and 4.

**Special Rates** will be quoted for those institutions that agree to send all their vacancy, contract, and public notices to The Hospital each year, so as to make it a file of such announcements for ready reference.

**Remittances:**

**It is especially requested that remittances be made by Cheque or Postal Order, and in halfpenny stamps only when the amount is under sixpence.**

